# Enhancing cochlear duct length estimation by incorporating second-turn parameters

**DOI:** 10.1038/s41598-023-48641-0

**Published:** 2023-12-06

**Authors:** Asma Alahmadi, Yassin Abdelsamad, Anandhan Dhanasingh, Fida Almuhawas, Abdulrahman Alsanosi

**Affiliations:** 1 King Abdullah Ear Specialist Center (KAESC), King Saud University Medical City (KSUMC), College of Medicine, King Saud University, P.O. Box: 245, 11411 Riyadh, Saudi Arabia; 2 Research Department, MED-EL GmbH, Riyadh, Saudi Arabia; 3 MED-EL Elektromedizinische Geraete Gesellschaft m.b.H., Innsbruck, Austria

**Keywords:** Surgery, Three-dimensional imaging, Anatomy, Medical research, Signs and symptoms

## Abstract

Estimating insertion depth, cochlear duct length (CDL), and other inner ear parameters is vital to optimizing cochlear implantation outcomes. Most current formulas use only the basal turn dimensions for CDL prediction. In this study, we investigated the importance of the second turn parameters in estimating CDL. Two experienced neuro-otologists blindly used segmentation software to measure (in mm) cochlear parameters, including basal turn diameter (A), basal turn width (B), second-turn diameter (A2), second-turn width (B2), CDL, first-turn length, and second-turn length (STL). These readings were taken from 33 computed tomography (CT) images of temporal bones from anatomically normal ears. We constructed regression models using A, B, A2, and B2 values fitted to CDL, two-turn length, and five-fold cross-validation to ensure model validity. CDL, A value, and STL were longer in males than in females. The mean B2/A2 ratio was 0.91 ± 0.06. Adding A2 and B2 values improved CDL prediction accuracy to 86.11%. Therefore, we propose a new formula for more accurate CDL estimation using A, B, A2, and B2 values. In conclusion, the findings of this study revealed a notable improvement in the prediction of two-turn length (2TL), and CDL by clinically appreciable margins upon adding A2 and B2 values to the prediction formulas.

## Introduction

Sensorineural hearing loss (SNHL) is the most frequent form of permanent hearing impairment and has an estimated prevalence of 0.13% among neonates in developed countries. In adolescents, this prevalence increases to 0.35% due to accumulated late-onset genetic and acquired cases^1,2^. Since the implementation of universal neonatal screening programs in most developed countries, patients with SNHL have been able to benefit from early intervention^3,4^. The gold standard for most cases of SNHL is the cochlear implant (CI). CIs are a safe and cost-effective treatment option for patients with severe-to-profound SNHL who derive insufficient benefit from hearing aids. CI use is an effective means of enabling patients to develop (or regain, in cases of post-lingual SNHL) hearing and communication abilities^5,6^.

For optimal outcomes, CIs must be tailored to each recipient's requirements. Cochlear shape, size, and length vary among individuals; this geometrical variation can reach up to 30–40%^7,8^. Advancements in CI are moving toward offering (electrode) arrays of varying lengths to account for individual variations in CDL, rather than using “one size” arrays designed for cochleae of average size. Such individualization can be used to maximize treatment outcomes and improve users’ quality of life. The intracochlear electrode array is placed adjacent to peripheral dendrites of the afferent auditory nerve and spiral ganglion cell bodies (SGCBs) in the scala tympani. Recently, Dhanasingh et al.^9,10^ demonstrated that SGCBs are distributed in the basal turn and extend into the second turn as well, with segment I (6 mm along the organ of Corti; angular depth, 75°) containing only 11.3% of SGCBs and segments II and III (15 mm from segment I; angular depth, up to 400°) containing 63%. However, segment IV (angular depth of up to 680°) contains 25.8% of SGCBs. There is a need to be precise in determining the balance between safe atraumatic insertion and optimum insertion depth of the array to maximize the coverage of the cochlea while preserving the hearing structures, thereby maximizing CI outcomes.

A deeper understanding and knowledge of cochlear architecture and morphology, and concise measurements of cochlear parameters will aid in better pre-operative estimation of cochlear duct length (CDL) and angular insertion depth. This mitigates the effects of incomplete array insertion, migration, folding, or kinking. Furthermore, it will yield a more precise allocation of frequency bands to electrode contacts, thereby producing maximum benefits and performance^11^. Currently, the most clinically viable methods to estimate the CDL and angular insertion depth are dependent only on cochlear basal turn parameters, which can be measured by different methods described in the literature^12–15^. The basal turn of the cochlea, while readily identifiable, does not provide a comprehensive representation of the cochlear structure due to its highly variable nature among individuals^16^. This is particularly critical for CIs, where an accurate assessment of CDL can influence array choice and placement, ultimately impacting hearing outcomes^17^. Furthermore, the middle turn contains a large fraction of the SGCBs, which play a pivotal role in CI stimulation and overall auditory function^18^. As a result, incorporating the second-turn parameters in CDL estimation could both lead to more accurate measurement and facilitate tailored approaches to CI, improving post-operative hearing performance. Thus far, no method has been developed to measure the second-turn parameters and incorporate them in the CDL. Moreover, the middle and apical turns are not always predicted by basal turn parameters^7^.

Although many CI centers use CDL preoperatively to select the array length that best fits the recipient’s cochlear architecture, there are still some CI cases with challenging and incomplete insertion or over-insertion. Thus, there is a need for a new method that more accurately estimate the CDL^11^. Although, sub-optimal cochlear implantation may not solely be due to inaccuracies in CDL prediction. Factors such as training and experience of the surgeon as well as the characteristics of the array itself play important roles in the success of the insertion process.

Therefore, this study aimed to investigate the importance of the second-turn parameters in estimating CDL. The secondary objective was to test the accuracy of our new proposed formulas in predicting more accurate CDL and 2TL.

## Methods

### Study design

This retrospective study was conducted at a tertiary CI center. Pre-operative CT scans of temporal bones were retrieved from the medical image database of King Abdullah Ear Specialist Center, King Saud University. All CT scans that showed a good compatibility with the 3D reconstruction software and resulted in a clear and high-resolution 3D model have been included to the study regardless age, gender, side. Patients were excluded if they had poor CT image resolution, in case of any inner ear anomalies, or if they had middle or inner ear pathologies. This study was conducted in accordance with the Declaration of Helsinki and approved by the local institutional review board of King Saud University (No. E-22-6574). The same local committee waived the need for informed consent due to the retrospective nature of the study. Routine clinical data, including age, sex, ear side, and array type, were collected.

### CT Measurements and data collection

All CT images in the present study were obtained using a 512-slice multidetector-row CT scanner (General Electric Healthcare). The following scanning parameters were used: axial plane, 0.625 mm slice thickness, 230 mAs, 140 kV, and rotation time 1 s with 0.3 mm reconstruction in the axial and coronal views. DICOM images of patients’ temporal bones were imported and segmented using the 3D Slicer multi-platform software (https://www.slicer.org/) following the previously reported steps^11^. The software’s four-window layout allowed us to manipulate and examine the image in axial, coronal, sagittal, and 3D segmentation views, the model was freely manipulated to allow for optimal visualization. The slice plane was adjusted parallel to and over the 3D segmentation, providing a consistent perspective for measurements. To standardize this approach, the 3D model was projected into a Cartesian coordinate system, with the X, Y, and Z axes set perpendicular to the cochlear aperture and the modiolus.

Two experienced neuro-otologists made the 3D segmentation and then measured the cochlear parameters in the oblique coronal plane for all patients. We extracted and reoriented 3D images into an oblique coronal plane to consistently define the “cochlear view”. This plane was carefully chosen to display the basal turn of the cochlea at its widest point, thereby enabling accurate measurements of the A and B values.

The lateral wall was identified as the outermost part of the cochlea in the oblique coronal plane of the CT images. We enhanced the visibility of this structure by maximizing the contrast between the bone and surrounding structures. The cochlear apex was determined by tracing the lateral wall of the cochlea from the round window to the point where the two turns of the cochlea could no longer be differentiated. This point was designated as the cochlear apex and was cross-verified on the axial plane. Recognizing that the angular length of cochleae is highly variable and can affect the length estimates using A and B values, we employed meticulous care in this identification process.

To achieve consistency, all segmentations and measurements were performed by the same two experienced neuro-otologists blindly, and the discrepancy between both readings were analyzed and tested. In instances where there was more than a 10% discrepancy between their measurements, a consensus meeting was held to discuss and re-examine the respective measurements.

**Figure 1:**
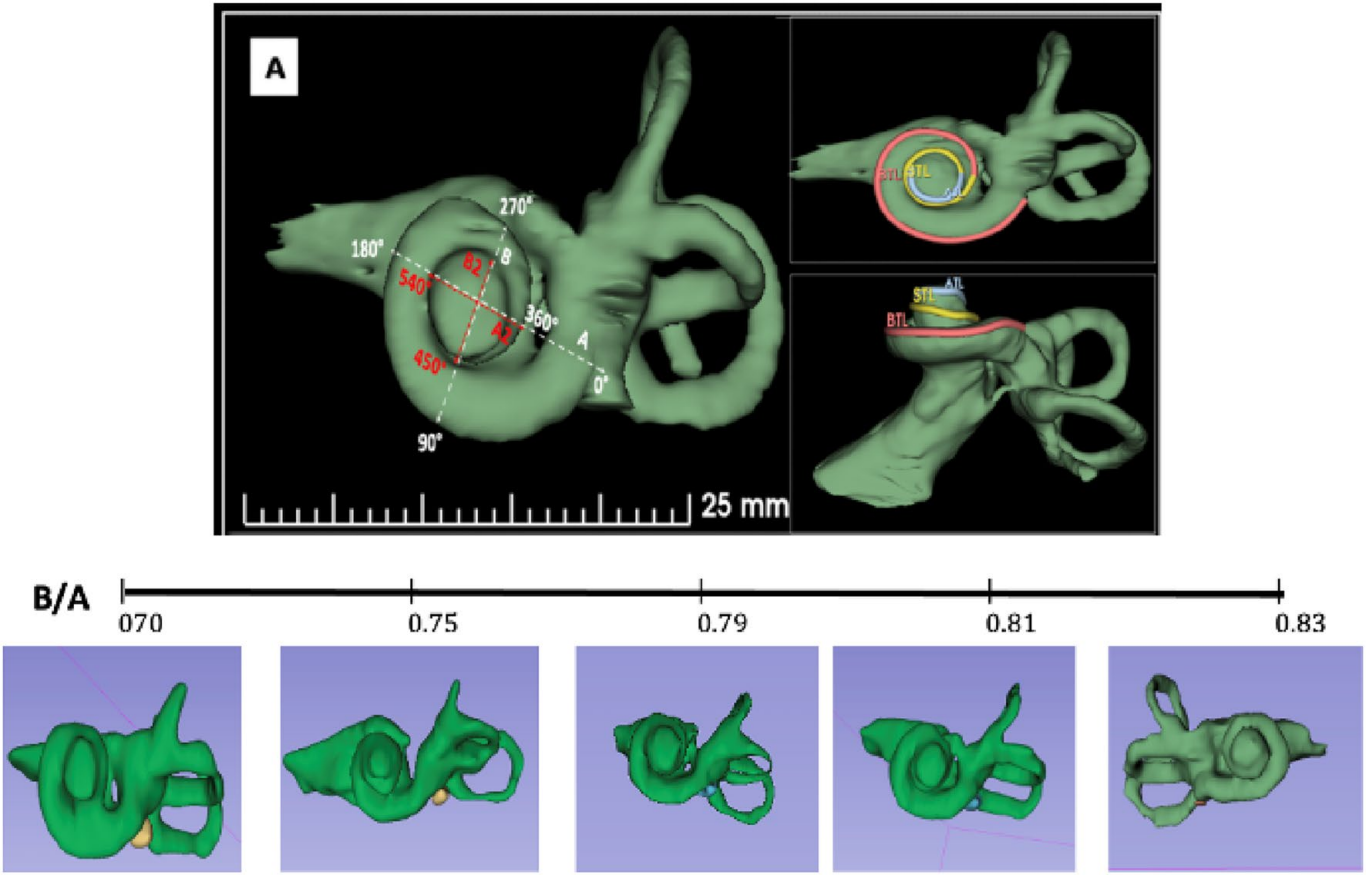
Graphical representation of the cochlea. The cochlear measurement is shown by ‘A’ where the white dashed line demonstrates the basal turn dimensions, the red lines demonstrate the second-turn dimensions, A is the diameter of the basal turn, B is the width of the basal turn, A2 is the diameter of the second turn, and B2 is the width of the second-turn. The variation in the basal turn shown by ‘B’ from more oval on the left to more rounded on the right due to variation in the B/A ratio. Simultaneously, the second turn is more frequently rounded owing to the high B2/A2 ratio.

We proposed the following steps to perform the measurements (Fig. 1):

The A and B values were measured in the cochlear view projection, as previously described by Escude et al.^14^. A line was drawn from the center of the round window passing through the central axis of the cochlea to a distant point of each turn and was used as a reference to start the measurement^19^. The A value (diameter) represented the largest linear distance of the basal turn from the center of the round window to the farthest point on the opposite of the lateral wall, passing through the modiolus. The B value (width) represented the width of the basal turn of the cochlea, measured as a straight-line running perpendicular to the A value line at the modiolus. We defined A2 as the diameter of the second turn, which was from the start of the second turn at 360° to the furthest point of the second turn through the modiolus. B2, which represented the width of the second turn, was measured as a line perpendicular to A2 that passed through the modiolus. CDL was measured manually by tracing a curve line from the center of the round window to the cochlear lateral wall up to the most apical bony point of the apex. The basal turn length (BTL) was measured manually as a curve line from 0° to 360° of the cochleae. Then, STL which was the second or mid-turn was measured as a curve line from 360° to 720° of the cochleae. Finally, the two-turn length (2TL) was both the basal and mid-turns of the cochlea from 0° to 720° of the cochleae.

The B/A ratio represented the ratio of the width to the diameter of the basal turn to determine the corresponding round/elliptical shape^8^. The B2/A2 ratio is introduced and used in this study to represent the ratio of the width to the diameter of the second turn to determine the cochlear shape.

### Statistical analyses

All statistical analyses were performed using Python version 3.9. Briefly, we determined the ICC between the two readers to validate their measurements. Parametric data are presented as mean ± SD, whereas nonparametric data are presented as median (Q1, Q3). For inferential statistics, we performed bivariate comparisons of the variables grouped by sex. We used the two-sided independent sample t-test for parametric data and the Mann–Whitney U test for nonparametric data. Furthermore, we used Pearson's correlation to estimate correlations between the B/A ratio to the B2/A2 ratio to assess cochlear dimensional consistency across patients.

To validate the importance of measuring A2 and B2 values for predicting CDL, and 2TL, we fitted the A, B, A2, and B2 values to predict them using multiple linear regression models. We compared these models to those fitted to the A and B values alone and to those fitted to A2 and B2 values alone. To further validate the results of these regression models, and to account for the small sample size, we performed a five-fold cross-validation and calculated the regression coefficients, mean R^2^, and test accuracy from the cross-validation for each model.

### Ethics declarations

The study was conducted in accordance with the Declaration of Helsinki and approved by the local institutional review board of King Saud University (No. E-22-6574).

### Informed consent

The aforementioned ethics committee waived the need for informed consent due to the retrospective nature of the study.

## Results

CT scans of 17 patients (33 ears) aged 1.6 (1.1–2.9) years with good resolution were eligible for the measurement of cochlear parameters. All implantations were with a MED-EL electrode array (Innsbruck, Austria): 15 ears received a FLEX28, 11 received a FORM24, 3 received a FLEX24, 3 received a STANDARD, and 1 received a MEDIUM. Table 1 shows the descriptive statistics of the patients. Female patients accounted for 45.5% of the study participants. A value, CDL, and first-turn length (BTL) were significantly shorter in females than in males (Table 1). BTL represented approximately 61% of the CDL, whereas STL represented only 32%.

**Table 1:** Descriptive statistics of study participants.

	Overall	Sex		
		Female	Male	p
n	33	15	18	–
Age (years), median [Q1, Q3]	1.6 [1.1, 2.9]	1.5 [0.9, 4.0]	1.6 [1.3, 2.7]	0.856
A, mean (SD)	8.4 (0.4)	8.2 (0.4)	8.6 (0.4)	**0.007**
B, mean (SD)	6.5 (0.4)	6.4 (0.3)	6.6 (0.4)	0.191
A2, median [Q1, Q3]	3.8 [3.6, 3.8]	3.7 [3.6, 3.8]	3.8 [3.6, 4.0]	0.466
B2, mean (SD)	3.4 (0.2)	3.4 (0.2)	3.5 (0.3)	0.323
CDL, mean (SD)	33.5 (1.6)	32.9 (1.3)	34.0 (1.7)	**0.048**
BTL, mean (SD)	20.6 (0.9)	20.2 (0.8)	20.8 (1.0)	**0.047**
STL, median [Q1, Q3]	10.4 [10.3, 11.1]	10.4 [10.2, 10.7]	10.7 [10.3, 11.1]	0.084

Data are presented as mean (standard deviation), median [Q1, Q3], or number (percentage). Continuous variables were grouped by sex and insertion and were tested for significant differences. The t-test was used for parametric variables, whereas the Mann–Whitney U test was used for nonparametric variables. p values < 0.05 are shown in bold.

We calculated the intraclass correlation coefficient (ICC) across the two measurements done by the two readers. A, A2, and B2 values showed ICC values consistently above 0.6, which were always significant (average fixed raters' ICC = 0.861, 0.766, and 0.771, respectively; p < 0.05). However, the B value showed moderate ICC values, which were also significant (average fixed raters' ICC = 0.450, p < 0.05). When assessing normality, only age, A2 value, and STL variables were not normally distributed (p < 0.001, p = 0.017, and p = 0.023, respectively); therefore, nonparametric tests were used to test those variables going forward.

**Figure 2:**
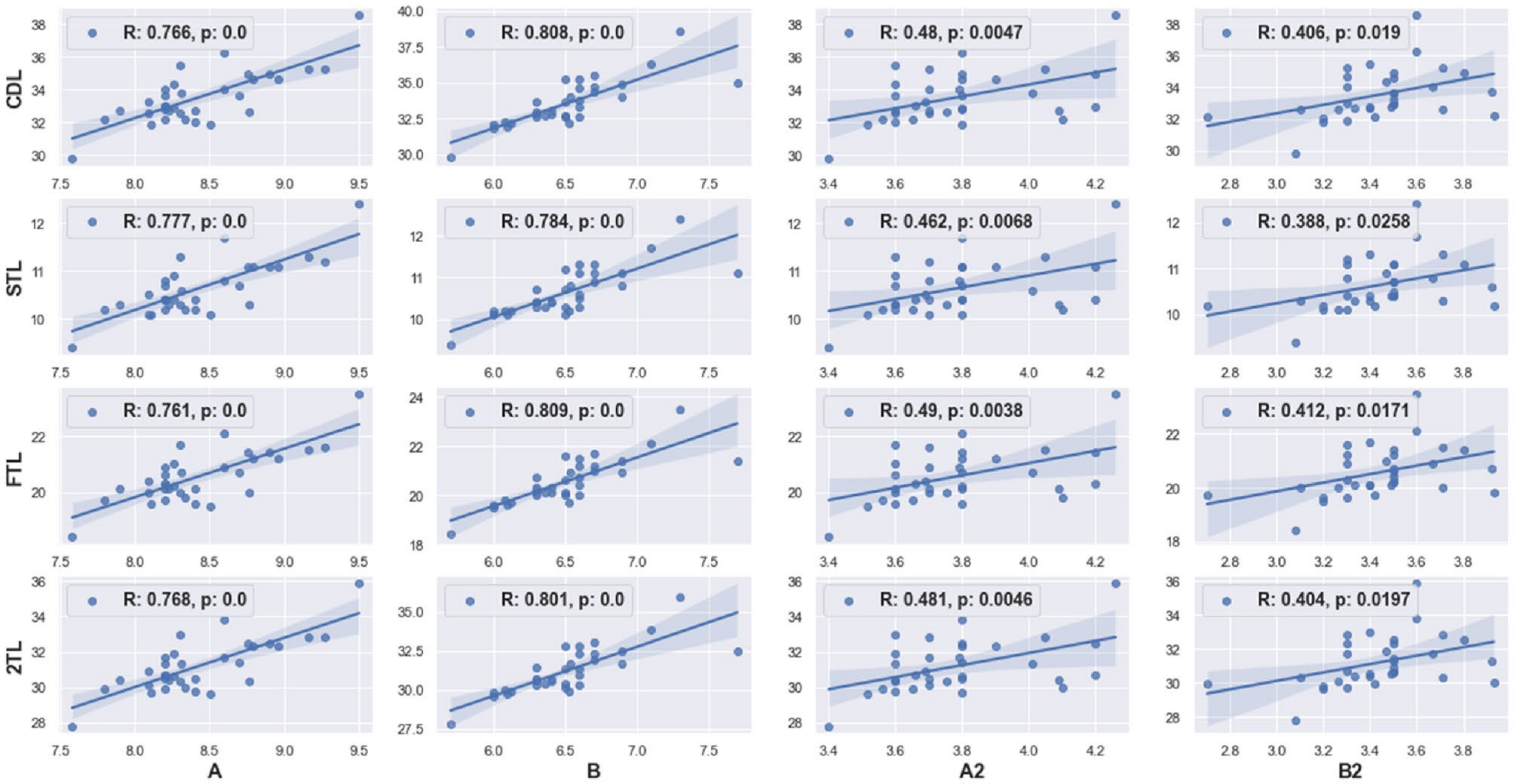
Pairwise correlations among various linear cochlear parameters. Parameters were correlated using Pearson's correlation test. CDL, cochlear duct length; BTL, first-turn length; STL, second-turn length; 2TL, two-turn length.

The B/A ratio ranged from 0.70 to 0.87 (mean = 0.77, standard deviation [SD] = 0.04), whereas the B2/A2 ratio ranged from 0.76 to 0.99 (mean = 0.91, SD = 0.06). The B/A ratio was not significantly correlated with the B2/A2 ratio (r = –0.467, p = 0.222). In contrast, the A value exhibited a significant moderate positive correlation with B (r = 0.727, p = 0.002) and A2 (r = 0.631, p = 0.022). B showed a similar correlation with B2 (r = 0.686, p = 0.006), and A2 showed a similar correlation with B2 (r = 0.747, p = 0.001). Figure 2 shows the remaining pairwise correlations of the cochlear parameters.

**Figure 3:**
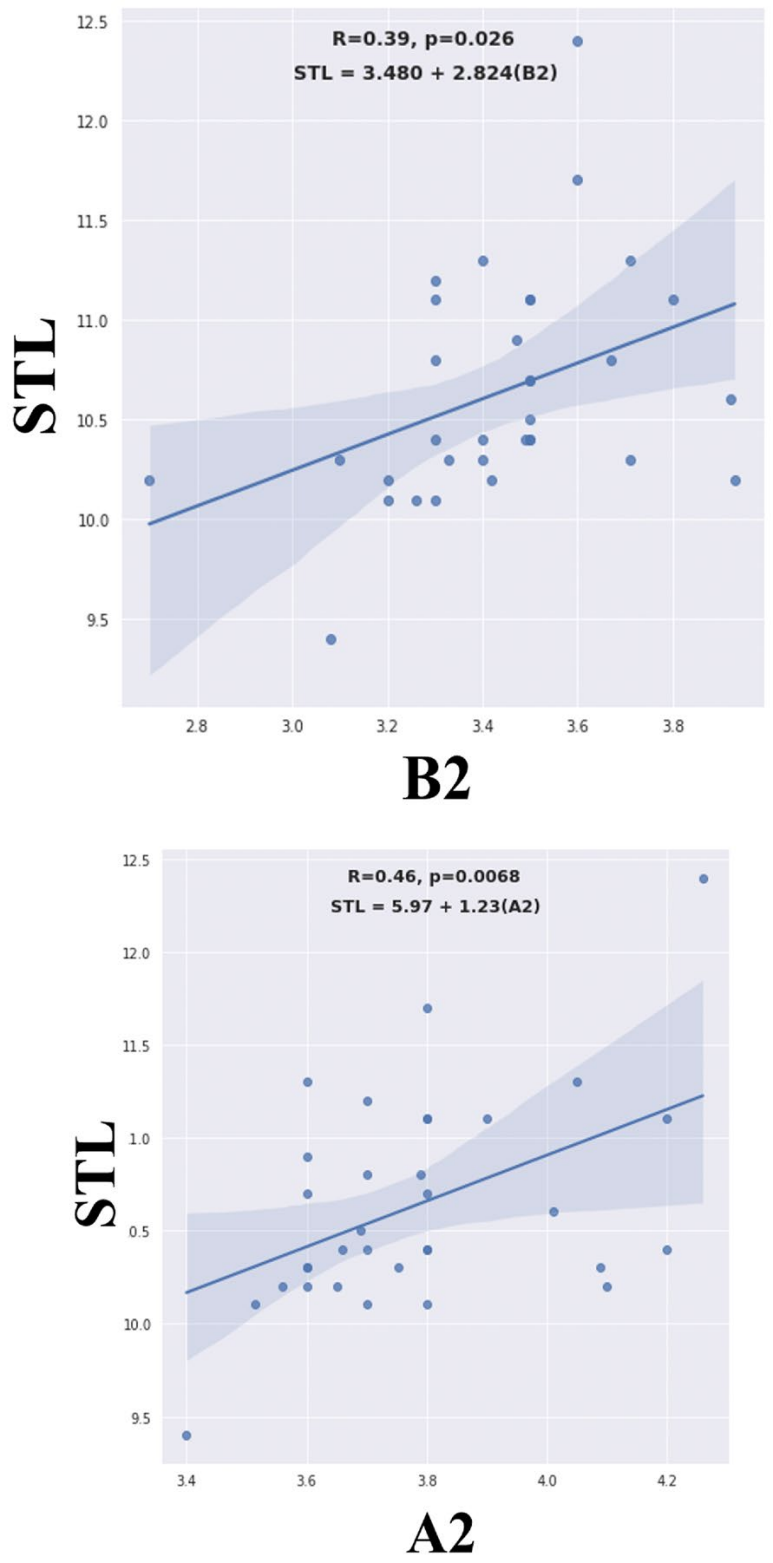
Relationship between length of second turn (STL) and “A2” and “B2”.

**Figure 4:**
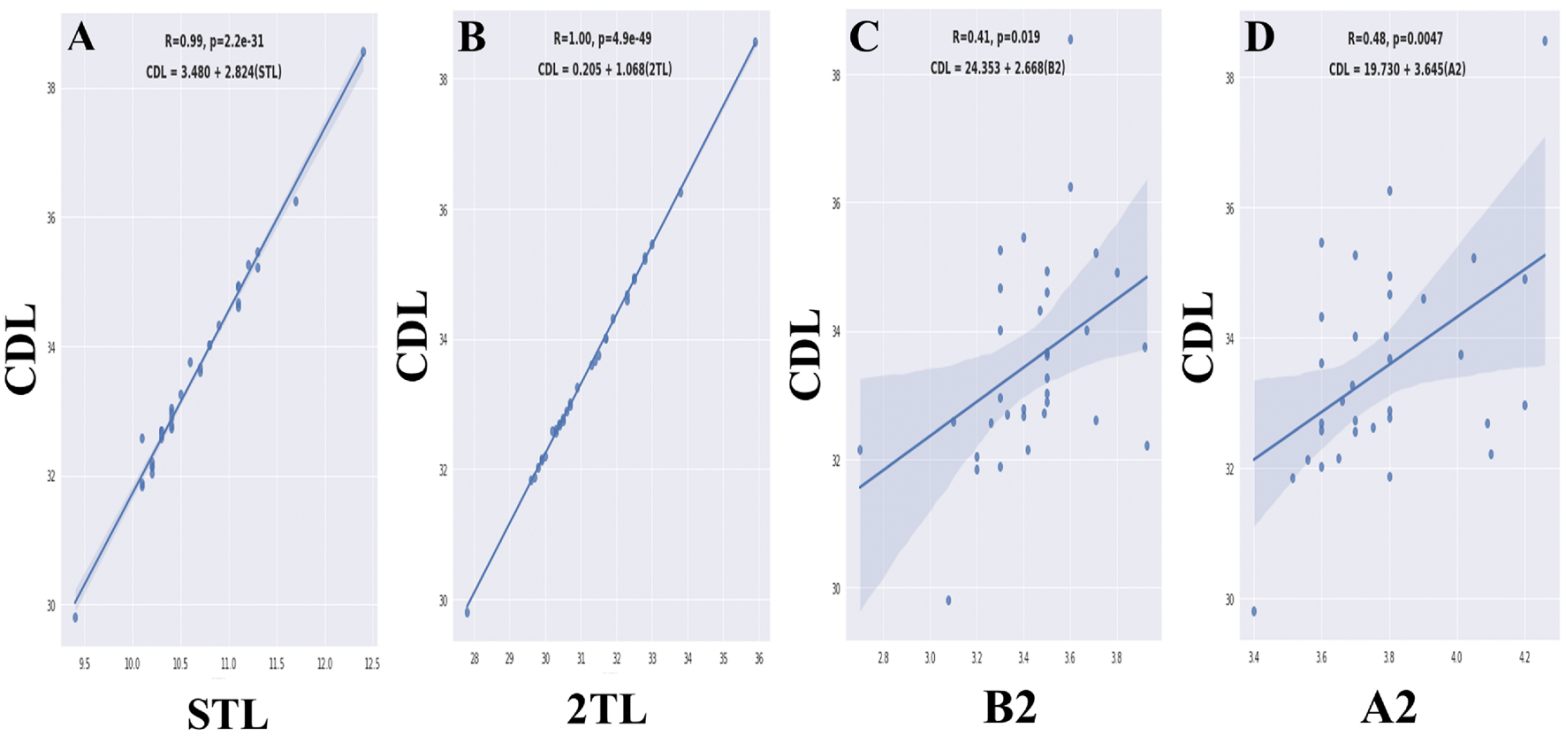
Relationship between cochlear duct length (CDL) and different measures. (**a**): relationship between CDL and the second-turn length (STL), (**b**): relationship between CDL and the two-turn length (2TL), (**c**): relationship between CDL and A2 values (length of second turn), (**d**): relationship between CDL and B2 values (width of the second turn).

The simple linear regression model showed that A2 has a significantly higher correlation (r = 0.46, p = 0.007) than B2 (r = 0.39, p = 0.026) with STL (Fig. 3):

The correlation of CDL with STL was 0.994 ($p < 0.001$), which was highly significant. For a 1-mm increase in STL, CDL increased significantly by 2.824 mm. The multiple $R^{2} = 0.988$ (Fig. 4a) shows that this is a very good fit.

From Fig. 4b, we observed that a 1 mm increase in the 2TL led to a significant increase of 1.068 mm ($p < 0.001$) in CDL. Multiple $R^{2} = 0.999$ indicated an almost perfect fit.

Figure 4 shows that CDL was significantly correlated with A2 values ($r = { }0.480,{ }p = { }0.005$). It also had a significant correlation with B2 values ($r = { }0.406,{ }p = { }0.019$).

When we used a multiple linear regression model with A2 and B2 values together, a significant fit was obtained with $R^{2} { } = { }0.209$ , and ($p = { }0.011$).

Next, to determine the effect of each variable in the regression models, we fitted a multiple linear regression model with the A, B, A2, and B2 values as predictors. We used five-fold cross-validation to estimate the test accuracy and model ${R}^{2}$ to assess the goodness of fit. Test accuracy was measured based on the correlation between the observed and predicted values for the test data. We found that adding A2 and B2 improved the test accuracy and increased the ${R}^{2}$ (a measure of training accuracy) for all models (Table 2). Therefore, we derived the Eqs. (1) and (2) that represent the fitted regression equations for predicting 2TL and CDL, respectively:

1 $${\text{2TL }} = { 1}.{\text{63A }} + { 2}.{\text{1B }} + \, 0.{\text{52A2 }} - \, 0.0{\text{1B2 }} + { 1}.{85}$$

2 $${\text{CDL }} = { 1}.{\text{71A }} + { 2}.{\text{29B }} + \, 0.{\text{54A2 }} - \, 0.00{\text{4B2 }} + { 2}.{17}$$

**Table 2:** Multiple linear regression models fitted to 2TL, STL, and CDL.

Independent variables (X)	Dependent variable (y)	Model score (R^2^)	Test accuracy (%)	Regression coefficients	Intercept
[‘A’, ‘B’, ‘A2’, ‘B2’]	2TL	0.82	86.11	[1.63, 2.1, 0.52, – 0.01]	1.85
[‘A’, ‘B’]	2TL	0.81	82.91	[1.73, 2.14]	2.64
[‘A2', ‘B2’]	2TL	0.27	43.14	[2.64, 1.21]	17.05
[‘A’, ‘B’, ‘A2’, ‘B2’]	STL	0.82	84.88	[0.66, 0.75, 0.13, – 0.02]	– 0.26
[‘A’, ‘B’]	STL	0.80	82.29	[0.68, 0.76]	–0.08
[‘A2’, ‘B2’]	STL	0.25	39.64	[0.95, 0.44]	5.53
[‘A’, ‘B’, ‘A2’, ‘B2’]	CDL	0.82	86	[1.71, 2.29, 0.54, – 0.004]	2.17
[‘A’, ‘B’]	CDL	0.81	82.97	[1.82, 2.33]	3.01
[‘A2’, ‘B2’]	CDL	0.27	42.28	[2.8, 1.32]	18.42

For each of these dependent variables, three models were fitted using A, B, A2, B2, or all four independent variables. 2TL, two-turn length, equals first-turn length plus second-turn length; STL, second-turn length; CDL, cochlear duct length.

## Discussion

In this study, we highlighted the importance of measuring A2 and B2 cochlear parameters to estimate STL, 2TL, and CDL in a cohort of pediatric CI recipients with structurally normal cochleae. In our cohort, we found that females had smaller A values, CDL, and BTL but not STL than males. This finding is consistent with that reported in previous studies in the literature^14,20,21^ and with the results of other studies conducted on the same population. Khurayzi et al.^22^ found a mean CDL of 32.91 mm ± 1.78 mm, which was significantly higher in males than in females. Notably, European populations tend to have longer CDLs^21,23^, whereas Asian populations tend to have shorter CDLs^16,24^. Alanazi and Alzhrani^20^ reviewed different cochlear lengths in different populations, further supporting these claims. Remarkably, the cochlea reaches maturity before birth^20^; therefore the CDL does not change with age. Thus, it is safe to assume that the CDL of our population is generally shorter than that of the European population.

In our sample, BTL mean and range (20.6 mm; 18.4–23.5 mm) represent 61% of the total cochlear length, whereas STL mean and range (10.6 mm; 9.4–12.4) account for 32% of the cochlear length, which was lower than the mean lengths for BTL (22.6 mm) and STL (12.4 mm) reported by Erixon et al.^25^. In addition, our B2 value (3.4 ± 0.2 [range, 2.7–3.93]) was lower than that mentioned by the same team (3.8 ± 0.25 [range, 3.3–4.3])^25^. These results suggest that cochlear size in our population is proportionally different from that of other populations. Additionally, Erixon et al. made their measurements on temporal bone corrosion casts, which provide a high level of detail and accuracy in capturing cochlear dimensions. In contrast, our study relied on 3D segmentation of clinical CT images. While CT imaging provides valuable insights into cochlear anatomy, it may introduce certain limitations compared to direct measurements on corrosion casts.

In our study, although the B/A ratio was not significantly correlated with the B2/A2 ratio, A and B showed moderate correlation with A2 and B2, respectively. This indicates significant morphological variability that is constrained by the linear lengths and widths in the studied cohort. Notably, compared with the basal turn, which can have an oval or round shape according to the B/A range as demonstrated by Khurayzi et al.^8^, we found that the second turn tends to be more frequently round, regardless of the shape of the basal turn in the same cochlea, due to the high B2/A2 ratio and significantly small variation (SD = 0.06). To explore these relationships, we might require a larger sample size with more variations in B2/A2 ratios to study the shapes of the second turn in depth.

Regression analyses showed that A2 or B2 can be used to predict STL, with A2 being the better predictor: for each 1 mm increase in A2 and B2 values, the STL increased significantly by approximately 1.23 and 0.90 mm, respectively. Further, adding the A2 and B2 values to our regression models improved the prediction accuracy for 2TL and CDL by approximately 3%. However, fitting regression models to a small sample size produces inconsistent results. Additionally, running the model several times can produce markedly different results, which means that A2 and B2 can be as important for CDL prediction, which might be revealed with a larger sample size.

We performed five-fold cross-validation across our models to minimize the inconsistency caused by the small sample size. Our analysis showed that the A and B values were consistently significant when predicting STL and CDL; the higher the A and B values, the higher the patients' CDL and STL. Surprisingly, these results did not extend to the A2 and B2 values, despite capturing features that should be associated with STL.

Various CI centers worldwide agree that most SGBCs are in the third and fourth cochlear segments^9,10^. Therefore, it is crucial to optimize array insertion depth to improve patient outcomes. Our results contribute to the body of evidence that can help surgeons increase the accuracy of CDL prediction.

Escudé et al.^14^ adapted the geometric modeling formula by Yoo et al.^26^, and used the A value and insertion angular depth to approximate the lateral wall length of the cochlea. They showed that the larger the A value, the higher the insertion angle depth and the longer the lateral wall length^14^. More recently, Schurzig et al.^15^ developed an elliptic-circular approximation algorithm to predict the insertion length and angles in each individual achieving higher accuracy estimation. The available equation-based models often include a predetermined representation of data based on the helical shape of the cochlea. Our study uses a validated multiple linear regression modeling approach; although the data representation is predetermined, it has more room to learn from the data provided, thereby producing predictions that fit the confounding population differences.

The process of calculating CDL and incorporating the second-turn parameters, A2 and B2, in the model is intricate, with a number of factors exerting influence. Firstly, the inherently variable nature of cochlear anatomy among individuals is one key consideration. Notably, the size, shape, and orientation of cochlear turns can vary greatly^27^, which influences the accuracy of A2 and B2 measurements, and by extension, the computation of 2TL, and CDL. Secondly, the technique used for measuring A2 and B2 can also affect the calculation. The precision of these measurements may be impacted by the resolution of the imaging technique, the angle at which measurements are taken, and potential observer variability in identifying anatomical landmarks^28^. Thirdly, our regression models assume a linear relationship between A2 and B2 values and cochlear lengths. This may not fully capture the complexity of the true relationship, particularly given the three-dimensional spiral structure of the cochlea^29^. Lastly, the demographic characteristics of the sample population, such as ethnic background, may affect the generalizability of the model and its prediction accuracy in different groups. Future research could focus on quantifying and minimizing the impact of these influencing factors, potentially through the use of automated measurement techniques, advanced imaging technologies, and stratified analysis according to demographic characteristics. Additionally, the use of more complex mathematical models may be warranted to reflect the intricate relationships more accurately between various parameters and cochlear lengths.

This study was limited by the relatively small sample size which might affect its generalization. Our measurements were taken from the lateral wall of the cochlea, which, while providing a practical and consistent landmark, does not necessarily correspond directly to the distance that the array would travel within the scala tympani during cochlear implantation. A radial correction factor to convert the cochlear length estimates from the lateral wall to the estimated array location within the scala tympani was not incorporated into our study.

We acknowledge that the potential for observer variability cannot be eliminated and may influence the computation of various lengths. Future work with bigger sample size should consider conducting an error analysis to quantify variability in slice angles and how such variation might affect measurements of A2 and B2, and ultimately the calculated lengths.

## Conclusion

We found that the second turn of the cochlea tends to be round more frequently regardless of the basal turn shape. The findings of this study revealed a notable improvement in the estimation of 2TL, and CDL by clinically appreciable margins upon adding A2 and B2 values to the prediction formulas. Thus, our model and formulas may help clinicians choose the proper electrode-array length before the CI surgery. This improvement comes at little to no cost and may be detrimental to some patient outcomes.

## Data Availability

The datasets used and analyzed during the current study are available from the corresponding author on reasonable request.
